# Endoscopic Removal of Non-Eroding Gastric Bands Post Silastic Ring Vertical Gastroplasty Using Lumen-Apposing Metal Stents—a Case Series

**DOI:** 10.1007/s11695-026-08902-z

**Published:** 2026-08-17

**Authors:** Amir Luchtenstein, Relly Reicher, Shai Meron Eldar, Adam Abu-Abeid, Danit Dyan, Sigal Fishman, Mati Shnell

**Affiliations:** 1 Bariatric Endoscopy Unit, Department of Gastroenterology, Tel Aviv Sourasky Medical Center, Gray Faculty of Medicine & Health Sciences, Tel Aviv University, Tel Aviv, Israel; 2 Bariatric Center, Department of Surgery, Tel Aviv Sourasky Medical Center, Gray Faculty of Medicine & Health Sciences, Tel Aviv University, Tel Aviv, Israel

## Abstract

**Introduction:**

Endoscopic removal of gastric bands after silastic ring vertical gastroplasty (SRVG) has emerged as a viable alternative to surgical revision. Most endoscopic removals require prior spontaneous band erosion into the gastric lumen. For non-eroded bands, self-expanding metal/plastic stents (SEMS/SEPS) have been used to induce erosion but carry risk of migration. We hypothesized that lumen-apposing metal stents (LAMS), with their antimigration flanged design, could safely induce controlled erosion with fewer complications. This study evaluates the feasibility and short-term outcomes of LAMS-assisted band removal.

**Methods:**

We retrospectively analyzed 10 patients with non-eroding silastic bands and obstructive symptoms (emesis, dysphagia, or regurgitation) who underwent endoscopic band removal using LAMS. LAMS placement was performed to induce controlled erosion and migration of the band into the gastric lumen. A second endoscopy was scheduled at 1–3 months to remove the stent and band. Clinical success was defined as successful band removal without periprocedural complications.

**Results:**

All 10 patients underwent successful endoscopic band removal without periprocedural complications and were discharged within 24 h. Four patients (40%) required additional endoscopic balloon dilatations for gastric stenosis. Emesis, reported preoperatively in all 10 patients (100%), was resolved in 8 patients (80%) following the procedure. Mean Eckardt symptom score improved from 4.3 ± 1.8 to 1.4 ± 2.2.

**Conclusion:**

LAMS-induced erosion is a feasible and technically successful endoscopic technique for removal of non-eroding gastric bands in SRVG patients, with a favorable short-term complication profile.

**Key Points:**

• LAMS achieved 100% technical success for non-eroding band removal (*n* = 10).

• No periprocedural complications; all patients discharged within 24 h.

• Emesis resolved in 80%; mean Eckardt score improved from 4.3 to 1.4.

• LAMS offers a minimally invasive alternative to surgical band revision.

## Introduction

Silastic ring vertical gastroplasty (SRVG), a variant of vertical banded gastroplasty (VBG) first described by Mason in 1982, was designed to induce weight loss through creation of a small gastric pouch with fixed outlet restriction [1, 2]. In SRVG, a small vertical gastric pouch is fashioned along the lesser curvature and reinforced with a non-expandable silastic ring to prevent stoma dilation. The operation was traditionally performed via open or mini-laparotomy approach, with some centers incorporating a Roux-en-Y gastroenterostomy for additional malabsorptive benefit [3, 4].

SRVG gained considerable popularity throughout the 1980s and 1990s as a restrictive bariatric procedure for clinically severe obesity before being largely replaced by laparoscopic adjustable gastric banding (AGB) and other minimally invasive approaches in the late 1990s and early 2000s [5].

Short-term complications included bleeding, abscess formation, staple-line leaks, vomiting, and wound infection [4, 6, 7]. Long-term complications were frequent and predominantly related to outlet stenosis, including vomiting, esophagitis, and staple-line disruption. Additional adverse events included incisional hernia, band migration, and nutritional deficiencies. Reoperation rates have been reported between 29% and 50% [8–11].

Reported band-removal rates range from 5% to 19% [8, 9, 12, 13], typically prompted by obstructive symptoms including stenosis or band erosion. In cases of band-removal the interval from initial SRVG to band-removal varies considerably, most often spanning several years [8, 14]. Surgical removal, traditionally performed via laparoscopy, can be technically challenging, particularly in the presence of adhesions or erosion, and carries risks of bleeding, infection, or staple-line disruption. Some patients require revisional bariatric surgery for recurrent obesity or persistent obstructive symptoms [5, 8, 15–17].

With recent advances in therapeutic endoscopy, endoscopic removal has emerged as a less invasive alternative, though applications have been largely limited to eroded bands [18, 19]. For non-eroded bands, self-expandable plastic or metallic stents (SEPS/SEMS) have been reported to induce erosion. However, their use carries risk of adverse events including stent migration and patient intolerance [20].

The lumen-apposing metal stent (LAMS), originally designed for transluminal drainage of pancreatic fluid collections and other applications, has expanded utility in therapeutic endoscopy [21–23]. The LAMS (Spaxus, Taewoong, South Korea; used in this study in the 16 mm × 20 mm configuration) is a fully covered, braided nitinol stent with bilateral saddle-shaped lumen-apposing flanges designed to anchor tissue on both sides and prevent migration. Its dumbbell shape provides a stable, self-retaining position across a target tissue interface, while its large-bore lumen maintains luminal patency. The erosion mechanism induced by the LAMS is thought to be primarily pressure-mediated: the radial force exerted by the lumen-apposing flanges against the silastic band results in progressive ischemic necrosis of the overlying tissue, ultimately facilitating intraluminal migration of the band. Mechanical abrasion from the stent surface may additionally contribute. Based on these properties, we hypothesized that LAMS placement could safely induce controlled erosion of non-eroding gastric bands while minimizing migration and other adverse events associated with conventional SEMS/SEPS.

The aim of this study was to evaluate the feasibility, technical efficacy, clinical effectiveness, and safety of LAMS-induced band erosion for endoscopic removal of non-eroding gastric bands in SRVG patients.

## Materials and Methods

### Study Design and Patient Population

**Fig. 1:**
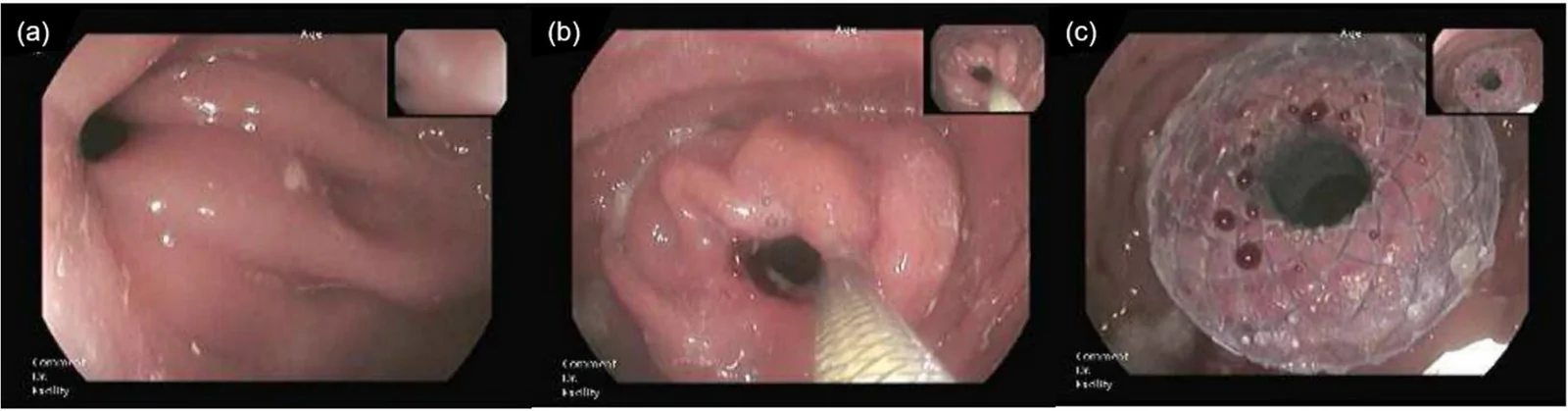
Endoscopic placement of a lumen-apposing metal stent (LAMS) across a non-eroded silastic band. (**a**) Gastric outlet stenosis caused by the ring. (**b**) Stent insertion across the stenosis. (**c**) Open LAMS within the gastric stenosis

We retrospectively analyzed consecutive patients referred to our center between 2022 and 2024 with obstructive symptoms (emesis, dysphagia, gastroesophageal reflux disease or regurgitation) attributed to pouch outlet stenosis and a history of SRVG (Fig. 1a). Initial diagnostic endoscopy was performed to assess band erosion, dilated pouch, staple line disruption as well as an alternative cause of the symptoms. Patients with significant malnutrition or comorbidities precluding safe endoscopy were excluded.

### Statistical Analysis

Descriptive statistics were used to summarize patient demographics, clinical characteristics, and outcomes. Values for continuous variables were calculated and expressed as mean ± standard deviation. Frequencies and percentages were reported for categorical variables. All analyses and results are presented in the tables below. Data were statistically analyzed using SPSS 26.0 (IBM Corp., Armonk, NY).

### Endoscopic Intervention

**Fig. 2:**
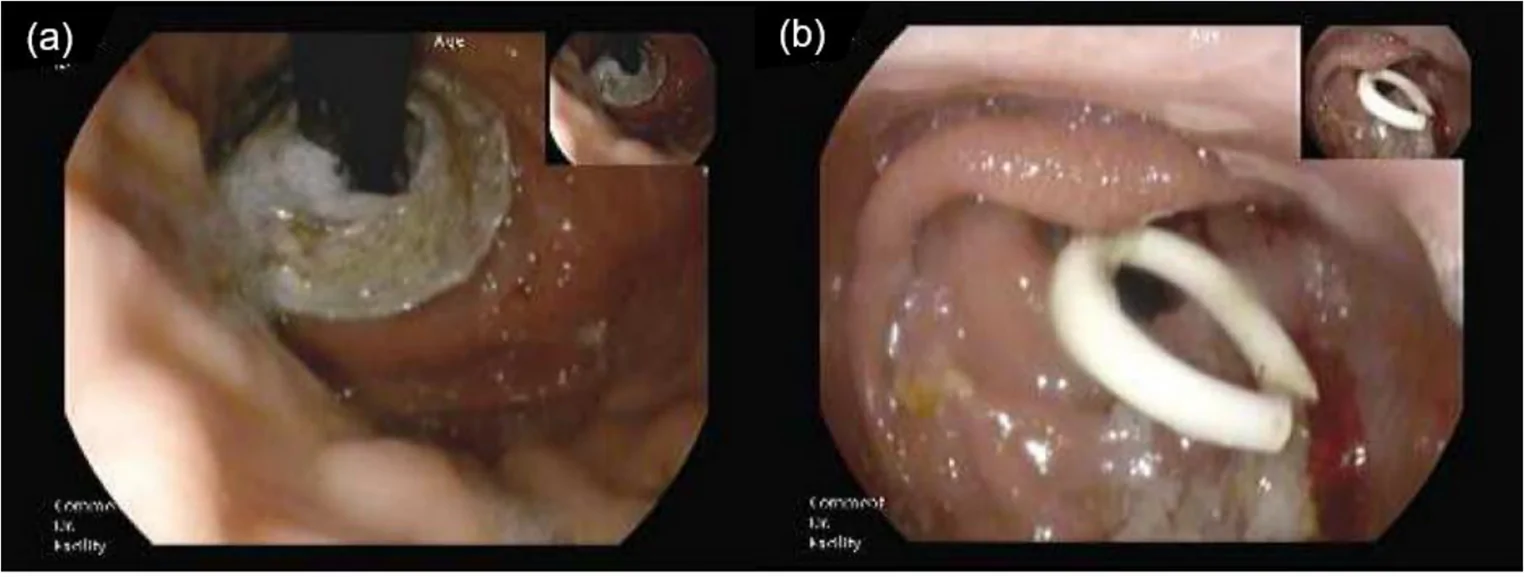
(**a**) Retroversion view of the placed LAMS two months after insertion. (**b**) Exposed silastic ring after stent removal

All therapeutic endoscopies were performed under conscious sedation in an outpatient setting. Carbon dioxide insufflation was employed throughout the procedure. The LAMS (Spaxus 16 mm × 20 mm, Taewoong, South Korea) was deployed across the band over a guidewire under endoscopic and fluoroscopic guidance. The correct position was assured by endoscopic visualization of the distal gastric body (Fig. 1b-c). The stent was left in situ to facilitate band erosion and migration into the gastric lumen. Patients were scheduled for follow-up endoscopy at 1–3 months for LAMS removal (Fig. 2a-b). If sufficient erosion was observed, the silastic band and the suture running through it were cut using endoscopic scissors (Ensizor, Slater endoscopy, USA), followed by complete removal with a grasper. A final inspection was performed to rule out adverse events such as bleeding or perforations. A routine proton pump inhibitor was prescribed for 1 month post-endoscopic treatment (Esomeprazole 40 mg BID). In cases where obstructive symptoms recurred after band removal, endoscopic dilation was performed using a 30 mm pneumatic balloon. The procedure was repeated if insufficient improvement was observed within 1–2 weeks.

### Outcomes Assessment

The primary endpoints were successful endoscopic procedures, defined as band erosion with complete band removal without additional surgical intervention, and clinical improvement, defined as improvement in patient-reported emesis and change in the Eckardt symptom score for achalasia before and after the procedure. Secondary endpoints included the rate of periprocedural and postprocedural complications, the need for further endoscopic treatment, and referral to other bariatric surgeries.

Primary outcomes included technical and clinical success. Technical success was defined as sufficient LAMS induced band erosion for complete endoscopic removal without the need for surgical intervention. Clinical success was defined as improvement in patient-reported emesis and change in the Eckardt symptom score for achalasia before and after the procedure. The Eckardt score was used to assess dysphagia for lack of a more specific dysphagia score following bariatric surgery (resolution of obstructive symptoms without periprocedural complications). Weight, as part of the Eckardt score, was measured at clinic visits using a calibrated scale and recorded in kilograms. Secondary outcomes included the rate of periprocedural and postprocedural complications, the need for further endoscopic treatment, and referral to other bariatric surgeries. All patients underwent postoperative follow-up by clinic visit or telephone contact for up to 4 months after removal to assess symptom resolution and weight trajectory, and to identify delayed complications. No patients were lost to follow-up.

## Results

Ten consecutive patients (9 women, 1 man) with a mean age of 60.7 ± 8.9 years underwent LAMS-induced band removal between 2022 and 2024 (Table 1). All patients were post-SRVG with symptomatic non-eroding bands. Time from initial SRVG to endoscopic band removal ranged from 23 to 32 years (mean 28.2 ± 2.7 years).

**Table 1:** Demographic and clinical characteristics of patients before stent placement

Characteristic	Value
Number of patients	10
Age, years (mean ± SD)	60.7 ± 8.9
Gender, n (%)	
Female	9 (90%)
Male	1 (10%)
BMI, kg/m² (mean ± SD)	31.6 ± 6.9
Duration with band, years (mean ± SD)	28.2 ± 2.7
Presenting symptoms, n (%)	
Emesis	10 (100%)
Dysphagia	8 (80%)
Eckardt score (mean ± SD)	4.3 ± 1.8
Endoscopic findings, n (%)	
Gastric stenosis	10 (100%)
Esophagitis	3 (30%)
Band erosion	0 (0%)

*BMI* body mass index,* SD* standard deviation

All 10 patients (100%) presented with emesis prior to intervention. Additional presenting symptoms included dysphagia (8 patients, 80%) and regurgitation (6 patients, 60%). Mean Eckardt symptom score was 4.3 ± 1.8. Esophagitis was noted in the initial endoscopy in 3 patients (30%).

LAMS placement was technically successful in all 10 patients (100% technical success). The mean interval between initial LAMS placement and follow-up endoscopy was 65.5 ± 16.7 days (range 36–91 days). At second endoscopy, the band had eroded and migrated into the gastric lumen in all cases, and both stent and band were successfully removed (Table 2).

**Table 2:** Procedural outcomes

Outcome	Value
Time with stent, days (mean ± SD)	65.5 ± 16.7
Erosion circumference after stent removal, % (mean)	86
Endoscopic success, n (%)	10 (100%)
Periprocedural complications, n (%)	0 (0%)
Delayed postprocedural complications, n (%)	1 (10%)
Balloon dilation, n (%)*	4 (40%)
Weight change since removal, kg (median, range)	3 (− 6 to + 25)

* Four patients had gastric stenosis after band removal that required balloon dilation

All patients experienced marked improvement of dysphagia and vomiting after stent insertion. Following the stent and band removal, symptoms recurred in 4 patients. In these patients, symptoms recurred within the first month post-removal, and re-stenosis was found in endoscopy. Endoscopic dilatation using a 30 mm pneumatic balloon (Rigiflex, Boston Scientific, USA) was performed. No adverse event was noted during endoscopic dilatation. Symptoms resolved in all patients, and no patient required surgical intervention.

At follow-up, emesis was resolved in 8 patients (80%) and improved but persisted in 2 patients (20%). Mean postoperative Eckardt symptom score improved to 1.4 ± 2.2, representing an overall improvement of 67% (Table 3). The median weight change since removal was an increase of 3 kg (range − 6 to + 25 kg).

**Table 3:** Clinical measures before and after ring removal

Emesis present, n (%)	Pre-procedure	Post-procedure
	10 (100%)	2 (20%)
Eckardt score (mean ± SD)	4.3 ± 1.8	1.4 ± 2.2

Periprocedural adverse events were absent in all cases. A single delayed postprocedural complication was reported in a patient with a mechanical mitral valve who was hospitalized two weeks after the procedure with suspected endocarditis and treated with antibiotics without the need for further intervention. No patient needed additional surgical intervention.

## Discussion

To our knowledge, this is the first case series describing use of LAMS for endoscopic removal of non-eroding gastric bands. Our findings suggest that LAMS-induced erosion represents a feasible and promising alternative to surgical band removal in patients with obstructive symptoms.

SRVG complications have been well described in the literature, with high rates of reoperation necessitated by obstructive symptoms and band-related erosion [8–11]. While surgical removal remains the standard approach, it carries significant morbidity including infection, bleeding, and staple-line disruption [17]. Endoscopic approaches for band removal have been reported, but prior techniques using conventional SEMS or SEPS have been limited by risk of stent migration and poor tolerability [18, 20, 24].

The lumen-apposing metal stent represents an important advance in therapeutic endoscopy, with expanding applications beyond its original use for pancreatic fluid collection drainage [21–23, 25]. The unique design of LAMS, with its lumen-apposing properties and antimigration flanges, may confer advantages over conventional fully covered self-expanding metal stents in this application. The stent geometry may facilitate controlled, localized erosion of the band with reduced risk of unintended migration or perforation. The LAMS used in this study was used off-label. As with all off-label uses of devices, relevant institutional and national regulations apply. Since its use for SRVG band removal is also off-label, it required informed consent. A similar LAMS device, such as the Axios stent (Boston Scientific), is FDA approved for drainage of pancreatic pseudocysts, walled-off necrosis, and gallbladder drainage in select patients, however it is frequently used for other off-label indications such as biliary drainage, gastroenterostomy, treatment of luminal strictures, and drainage of postsurgical collections.

In our series, all patients achieved successful band removal without periprocedural complications. The 40% rate of requiring additional balloon dilatation reflects the degree of fibrosis that can occur around bands over 23–32 years. This finding is consistent with the literature on late bariatric surgery complications and does not represent a failure of the technique but rather expected tissue response to long-standing foreign material [13, 15, 16]. Despite the high fibrosis rate, all balloon dilatations were successful clinically, and patients ultimately required no surgical management for stenosis. This emphasizes the importance of careful follow-up and may raise the question of a scheduled routine endoscopy 1–2 months after band removal. A proactive approach can help to achieve optimal patient outcomes.

The improvement in Eckardt symptom score and resolution of emesis in 80% of patients demonstrates meaningful clinical benefit. The two patients with persistent mild symptoms represent acceptable outcomes given the minimally invasive nature of the approach. Of note, both patients experienced weight regain following the treatment and refused further endoscopic dilatation. The expectation of weight regain following removal of a restrictive band is well-established, and patients should receive appropriate nutritional and bariatric counseling post-procedure.

All endoscopies were performed under conscious sedation without the need for intratracheal intubation. Both initial stent insertion and the subsequent stent and band removal were conducted in an outpatient setting, with patients able to be discharged the same day. The low complication rates and short hospital stays underscore the procedure’s safety and cost-effectiveness.

No adverse events were noted during the procedures and immediate follow-up. The suspected case of endocarditis in one patient with a mechanical mitral valve two weeks post-procedure warrants discussion. Although larger studies report no similar cases [19, 26], and ASGE/AHA guidelines do not recommend routine antibiotic prophylaxis for prosthetic heart valves during endoscopic procedures [27, 28], this case highlights the need for individualized risk assessment, prophylactic considerations, and vigilant post-procedural monitoring in high-risk patients.

Known LAMS complications, such as bleeding or perforation, were not observed in our cohort, nor did stent migration occur, which we attribute to the lumen-apposing flanged design of the LAMS. A buried stent, caused by tissue hyperplasia embedding the stent into the wall, is a delayed complication, mostly occurring with longer LAMS indwelling time. We restricted the indwelling to 3 months to reduce the risk of tissue overgrowth covering the stent. These potentially serious complications should be anticipated and monitored for in any larger-scale study employing this technique. A low threshold for early endoscopic re-evaluation in symptomatic patients is recommended, and endoscopic surveillance should be considered if a prolonged indwelling period is considered.

Our study has several limitations inherent to a small retrospective case series, including the absence of a control group. The variable interval from SRVG to intervention may have influenced symptom severity. Longer follow-up periods and a larger, prospective series would strengthen conclusions regarding the durability of symptom relief and long-term safety.

Future directions should include prospective comparative studies of LAMS-induced band erosion versus surgical removal, with longer follow-up to allow for identification of patient and technical factors predicting success or complications.

## Conclusion

LAMS-induced erosion is a feasible and technically successful endoscopic technique for removal of non-eroding gastric bands in SRVG patients. This approach offers a minimally invasive alternative to surgical revision with excellent technical success rates, a favorable short-term safety profile, and meaningful clinical benefit. LAMS-assisted band removal further expands the therapeutic endoscopic armamentarium for management of late SRVG complications. Given the small sample size and retrospective design of this case series, larger prospective studies are required to establish the long-term safety and efficacy of this technique.

## Data Availability

The datasets generated and analyzed during the current study are not publicly available due to patient privacy considerations but are available from the corresponding author on reasonable request.
